# Semi-Mechanistic Pharmacokinetic-Pharmacodynamic Model of Camostat Mesylate-Predicted Efficacy against SARS-CoV-2 in COVID-19

**DOI:** 10.1128/spectrum.02167-21

**Published:** 2022-04-12

**Authors:** Yuri Kosinsky, Kirill Peskov, Donald R. Stanski, Diana Wetmore, Joseph Vinetz

**Affiliations:** a M&S Decisions LLC, Moscow, Russia; b Sechenov First Moscow State Medical University, Moscow, Russia; c STU “Sirius,” Sochi, Russia; d NDA Partners LLC, Atherton, California, USA; e Harrington Discovery Institute, University Hospitals Cleveland Medical Center, Cleveland, Ohio, USA; f Section of Infectious Diseases, Department of Internal Medicine, Yale University School of Medicine, New Haven, Connecticut, USA; Wright State University

**Keywords:** COVID-19, antiviral pharmacology, camostat

## Abstract

The SARS-CoV-2 coronavirus, which causes COVID-19, uses a viral surface spike protein for host cell entry and the human cell-surface transmembrane serine protease, TMPRSS2, to process the spike protein. Camostat mesylate, an orally available and clinically used serine protease inhibitor, inhibits TMPRSS2, supporting clinical trials to investigate its use in COVID-19. A one-compartment pharmacokinetic (PK)/pharmacodynamic (PD) model for camostat and the active metabolite FOY-251 was developed, incorporating TMPRSS2 reversible covalent inhibition by FOY-251, and empirical equations linking TMPRSS2 inhibition of SARS-CoV-2 cell entry. The model predicts that 95% inhibition of TMPRSS2 is required for 50% inhibition of viral entry efficiency. For camostat 200 mg dosed four times daily, 90% inhibition of TMPRSS2 is predicted to occur but with only about 40% viral entry inhibition. For 3-fold higher camostat dosing, marginal improvement of viral entry rate inhibition, up to 54%, is predicted. Because respiratory tract viral load may be associated with negative outcome, even modestly reducing viral entry and respiratory tract viral load may reduce disease progression. This modeling also supports medicinal chemistry approaches to enhancing PK/PD and potency of the camostat molecule.

**IMPORTANCE** Strategies to repurpose already-approved drugs for the treatment of COVID-19 has been attractive since the beginning of the pandemic. Camostat mesylate, a serine protease inhibitor approved in Japan for the treatment of acute exacerbations of chronic pancreatitis, inhibits TMPRSS1, a host cell surface serine protease essential for SARS-CoV-2 viral entry. *In vitro* experiments provided data suggesting that camostat might be effective in the treatment of COVID-19. Multiple clinical trials were planned to test the hypothesis that camostat would be beneficial for treating COVID-19 (for example, clinicaltrials.gov, NCT04353284). The present work used a one-compartment pharmacokinetic (PK)/pharmacodynamic (PD) mathematical model for camostat and the active metabolite FOY-251, incorporating TMPRSS2 reversible covalent inhibition by FOY-251, and empirical equations linking TMPRSS2 inhibition of SARS-CoV-2 cell entry. This work is valuable to guide further development of camostat mesylate and possible medicinal chemistry derivatives for the treatment of COVID-19.

## INTRODUCTION

The coronavirus disease 2019 (COVID-19) pandemic has reinforced the need for early oral treatment to prevent disease progression (1). Currently such drugs are not available. Drug repurposing, based on a relevant mechanism of action as driving rationale is attractive (2, 3). Current treatment of COVID-19 is primarily hospital-based and directed at advanced disease, for example with remdesivir (which, despite FDA approval based on three pivotal trials [4–6]), or corticosteroids such as dexamethasone (7, 8). Monoclonal antibodies can be used in the outpatient setting but are expensive, logistically challenging to administer, and have variable degrees of efficacy due to viral variants (9).

Remdesivir, an inhibitor of the viral RNA-dependent, RNA polymerase, is the most widely investigated antiviral drug for the treatment of COVID-19 (10). In one clinical trial remdesivir was shown to reduce the time to recovery in patients who were hospitalized with COVID-19 (4). But in other trials remdesivir has not been shown to have a potent antiviral effect in moderate (5) and severe (6) COVID-19 patients.

Molnupiravir is a newer oral antiviral drug that has recently been tested in COVID-19 (11). Early treatment with molnupiravir reduced the risk of hospitalization or death in at-risk, unvaccinated adults with COVID-19 (12). However, its role in moderate to severe COVID-19 is questionable and more studies are needed. Particularly, in the phase 2 trial of patients hospitalized with COVID-19, a 5-day course of molnupiravir did not demonstrate clinical benefit (13).

Despite this recent progress with molnupiravir, additional therapeutic options are needed, particularly for early treatment of SARS-CoV-2 infection, i.e., newly diagnosed individuals in the outpatient setting, as well as for post-exposure prophylaxis.

**FIG 1:**
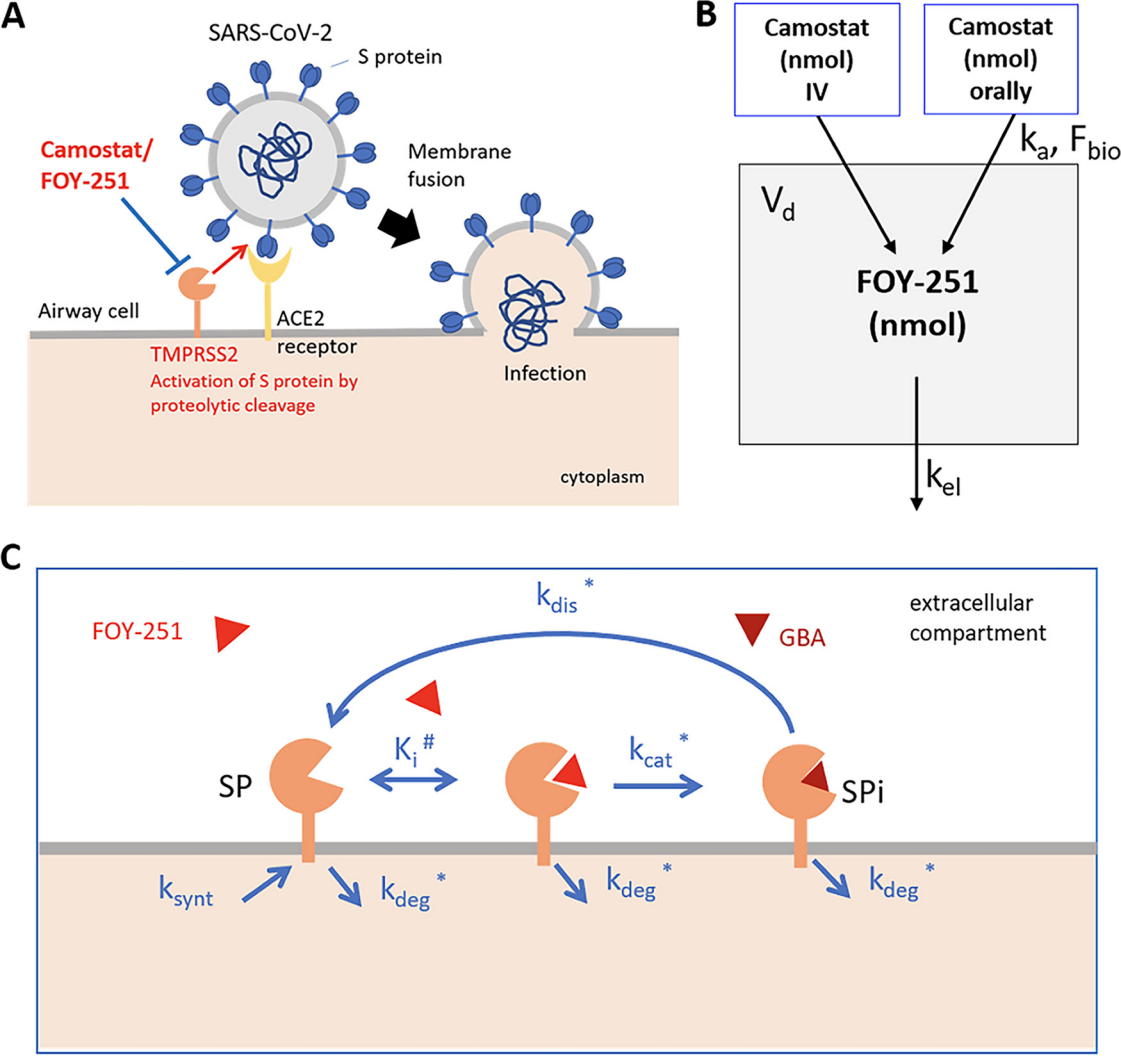
The model schemes. (A) Biological model of targeting SARS CoV-2 cell entry through the TMPRSS2 inhibition. (B) Pharmacokinetic model for Camostat/FOY-251. Very fast transformation of Camostat mesylate into the active metabolite FOY-251 in physiological fluids is assumed. (C) Pharmacodynamic TMPRSS2 inhibition model (*in vivo*). The model considers TMPRSS2 synthesis (k_synt_) and degradation rates (k_deg_). TMPRSS2 inhibition by FOY-251 is considered as two-stage process: (i) 374 reversible binding (parameter *K_i_*) of the drug, and (ii) the drug covalent binding to the target residue (k_cat_). The recovery of TMPRSS2 activity (with inactive metabolite [GBA] molecule release from active center) is described by parameter k_dis_ (half-life of SPi state is about 14 h). For TMPRSS2 *in vivo* model ksynt was calculated based on k_deg_ value(assumed) and SP baseline level = 1.

Previous experimental data (14, 15) has established that the SARS-CoV-2 spike (S) protein uses the host cell factors angiotensin-converting enzyme 2 (ACE2) to bind to target cells, and that the host cell surface transmembrane protease serine 2 (TMPRSS2) cleavage of S protein enables entry into target cells (Fig. 1A).

Camostat mesylate and the related molecule, nafamostat, are approved in some countries (but not in the United States) for the treatment of pancreatitis and esophagitis (16, 17). Both molecules block TMPRSS2 priming of S protein *in vitro*, a mechanism that has been shown to be both necessary and sufficient for viral entry into respiratory epithelial cells (14, 15, 18). Camostat and nafamostat was shown to block SARS-CoV-2 infection of primary human lung epithelial cells (18) and nafamostat was found to inhibit SARS-CoV-2 spread and pathogenesis in mice (19). Therefore, camostat mesylate, which has a favorable safety profile (20), might be suitable for prevention and treatment of COVID-19 and related viral diseases, because SARS-CoV, MERS-CoV and several influenza A viruses depend on TMPRSS2 for spread and pathogenesis (14). Camostat mesylate is a pro-drug that following delivery to the bloodstream, is rapidly converted to the pharmacologically active metabolite FOY-251 which exhibits the TMPRSS2 inhibition. FOY-251 inhibit SARS-CoV-2 infection in Calu-3 lung cells culture with EC50 of 178 nM (18).

Camostat mesylate is currently being investigated, at the clinical dose of 200 mg every 8 h, as a treatment of COVID-19 in several clinical trials in Denmark, Israel, and the United States (NCT04321096, NCT04353284, NCT04355052, NCT04374019, and others). The first clinical trial results of camostat in hospitalized COVID-19 patients have been recently published and, while significant adverse events were not observed, conclusions regarding efficacy appear to be negative (21, 22).

This study took a modeling approach to provide quantitative estimates, using published pharmacokinetic (PK)/pharmacodynamic (PD) data and experimentally inferred mechanism of action of camostat mesylate, of this compound’s anti-SARS-CoV-2 effects in humans.

## RESULTS

### Camostat mesylate pharmacokinetic model in humans.

The pharmacological properties of camostat have been studied in detail (23). Due to rapid esterase conversion of camostat to 4-(4-guanidinobenzoyloxy) phenylacetic acid (GBPA also called FOY-251) during and after the uptake from the gut, only FOY-251 but not camostat is detectable in plasma. Both camostat and FOY-251 are potent inhibitors of serine proteases, but the FOY-251 metabolite, guanidinobenzoic acid (GBA), is inactive. Because FOY-251 is the only driver of TMPRSS inhibition *in vivo*, we focused on FOY-251 pharmacokinetics for antiviral modeling.

Fig. 1B displays the scheme of a one compartment PK model that we used to capture oral and IV administration of camostat, the active metabolite FOY-251 distribution and elimination. Due to rapid transformation of camostat into FOY-251 *in vivo*, in the model camostat dose is considered as equimolar in amount to FOY-251.

Human data following a 12-h intravenous infusion (23) and human data after oral administration (from the FOIPAN package insert) were digitized and used to fit the PK model parameters. The model describes well the observed time courses of FOY-251 concentration after both intravenous and oral administration of camostat (Fig. S1). Table 1 summarizes the parameters of the PK model including the estimation uncertainty (relative standard errors, % R.S.E.) which is low and acceptable for the nature of the data. Camostat bioavailability (F_bio_) was estimated to be low, approximately 5%. The volume of distribution of FOY-251 was 22.4 liters, approximating plasma volume/extracellular water space with limited tissue distribution. The terminal elimination half-life of FOY-251 was estimated to be about 0.6 h. FOY-251 volume of distribution and elimination half-life agree with published estimates (23).

**TABLE 1:** The PK model parameters

Parameter	Value	R.S.E. (%)	Comments
k_a_, 1/h	0.67	5.78	Absorption rate
F_bio_	0.051	4.66	Camostat oral bioavailability
V_d_, L	22.36	7.07	Volume of distribution
k_el_, 1/h	1.22	4.70	Elimination rate
Residual error model			
b	0.14	17.3	Proportional residual error model parameter

### FOY-251 distribution between extracellular fluid and epithelial lining fluid in upper airways.

Because SARS-CoV-2 primary infection progresses from the upper respiratory tract epithelium to the bronchoalveolar epithelium, it is important to estimate the drug concentration in epithelial lining fluid (ELF) in airways (Fig. S2A). Small molecule drugs freely penetrate through the pores in the pulmonary capillary between plasma and the extracellular fluid. Alveolar epithelial cells are connected by tight junctions that potentially restricts passive diffusion between cells. To reach ELF a drug must pass through the alveolar epithelial cells themselves. The factors influencing how antibiotic drugs exposure in ELF are discussed in (24).

The pharmacokinetic profiles of an antibiotic drug (cefdinir) in plasma and in “blister fluid” (which approximates the ELF fluid) were shown in (24). Comparing physicochemical properties of cefdinir and FOY-251 we expect a qualitatively similar PK profile for FOY-251 in ELF. FOY-251 is more hydrophobic than cefdinir, and, thus, might have even better permeability through the cell membrane than cefdinir. Applying an empirical formula from (24) to FOY-251, drug exposure in the ELF compartment was predicted to be 80% that of plasma (versus 60% for cefdinir). Thus, similar FOY-251 exposure and anti-SARS-CoV-2 effect is expected in plasma/extracellular fluid and in ELF compartment, respectively. On the other hand, not only FOY-251 permeability but also degradation rate in ELF compartment (which is unknown) might limit the drug exposure.

### FOY-251 semi-mechanistic pharmacodynamic model based on *in vitro* data.

Camostat mesylate and its active metabolite FOY-251 are not only competitive inhibitors of TMPRSS2 and analogous serine proteases, but inhibit these enzymes via covalent binding with the serine-441 residue in the active site (25, 26). We explicitly considered reversible covalent inhibition of TMPRSS2 by FOY-251 in our model (Fig. 1C). This mechanism includes reversible non-covalent binding of the FOY-251 molecules (red triangle on the scheme) into the enzyme active site (*K_i_* parameter), and then covalent binding of the drug molecule with the serine protease (k_cat_ parameter). As it was measured for TMPRSS2 homolog enteropeptidase incubated with camostat, the covalent complex is relatively stable, but reversible, with release of GBA molecule from the enzyme active site (27). In the model, the enzyme activity recovery is described by the k_dis_ parameter whose value was fixed and corresponds to enzyme-drug covalent complex half-life of 14 h as estimated for enteropeptidase.

**FIG 2:**
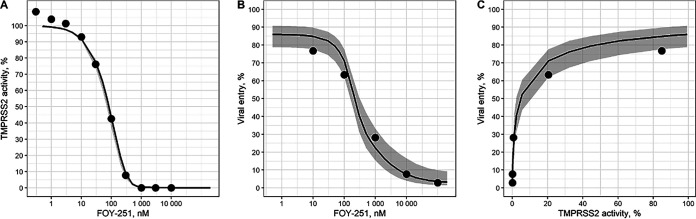
FOY-251 Pharmacodynamic models fitted to the data of *in vitro* experiments of M. Hoffman et al. (18). (A) Recombinant TMPRSS2 activity (relative to control) versus FOY-251 concentration, incubation time 1 h. (B) Viral entry rate (relative to control) versus FOY-251 concentration, incubation time 2 h. (C) Viral entry rate dependence (from panel B) on the respective model predicted TMPRSS2 activity, incubation time 2 h. The model predictions are shown by solid lines with 90% CIs (gray-color filled bars), the data from *in vitro* experiments (digitized from [18]) are shown by black filled circles.

Experimental data (18) were used to parameterize the PD model. First, the data of recombinant TMPRSS2 inhibition by FOY-251 after 1 h of incubation in a cell-free assay was considered. Fig. 2A depicts remaining enzymatic activity of the drug target enzyme TMPRSS2 against FOY-251 after incubation with FOY-251. The second experiment demonstrates dose-dependent inhibition of SARS-2-S-driven viral entry in a cell-based assay (Fig. 2B). The data of both experiments were combined, and the parameters of the *in vitro* PD model estimated with acceptable uncertainty (Table 2).

**TABLE 2:** The PD model parameters

Parameters	Value	R.S.E. (%)	Comments
TMPRSS2 activity inhibition			
k_cat_, 1/h	400		FOY-251 covalent binding to TMPRSS2 rate
K_i_, nM	45,638.51	7.46	FOY-251 inhibition constant
k_dis_, 1/h	0.049		TMRSS2 activity recovery rate after covalent inhibition by FOY-251
Viral entry rate inhibition			
K_sp_	0.047	42.4	TMPRSS2 activity corresponding to half-maximal viral entry rate
h	0.59	16.6	Hill coefficient in viral entry rate dependence on TMPRSS2 activity
Residual error model			
a_1_, %	3.46	22.5	Constant residual error model parameter for TMPRSS2 activity
a_2_, %	7.58	29.6	Constant residual error model parameter for Viral entry rate

Only k_cat/_*K_i_* model parameters ratio can be estimated from experimental data points. Thus, the parameter value k_cat_ = 400 1/h was fixed, and *K_i_* = 45.6 uM was estimated from experimental data. The assumptions made about k_cat_ and k_dis_ parameter values allows us to properly describe the data of both *in vitro* experiments (Fig. 2A, B).

The SARS-2-S-driven viral entry rate apparently depends on remaining TMPRSS2 activity as described by the Hill-Langmuir equation (see Materials and Methods), with TMPRSS2 activity producing half-maximal viral entry rate K_sp_ = 0.047 and Hill coefficient h = 0.59. This indicates that about 95% inhibition of TMPRSS2 is required for 50% inhibition of the rate of viral entry (Fig. 2C). The same parameter values were used to link TMPRSS2 inhibition with SARS-2-S-driven cell entry in *in vivo* PK/PD modeling.

### Camostat mesylate PK/PD model simulations in human.

The camostat/FOY-251 PK model predictions were used as input for mechanistic PD model predicting TMPRSS2 inhibition and respective SARS-2-S-driven viral entry rate inhibition. Rapid exchange between blood plasma and extracellular fluid compartments would be expected for small zwitterionic molecules like FOY-251. Thus, FOY-251 concentration in extracellular fluid of lung tissue is assumed to be equal to plasma concentration.

Fig. 1C displays the scheme of the *in vivo* semi-mechanistic PK/PD model. The TMPRSS2 molecules expressing on the cell surface of a respiratory epithelial cell are depicted in active and inactive states. The *in vivo* PK/PD model includes TMPRSS2 protein synthesis and degradation rates. Potentially, TMPRSS2 fast turnover rate might be a limiting factor for the drug effect, because all newly synthetized enzyme molecules are in the active state. No TMPRSS2 half-life estimate was found in the literature, and the assumed half-life 12 h was used to calculate the model parameter k_deg_ (=log (2)/T_1/2_ = 0.0575 1/h).

**FIG 3:**
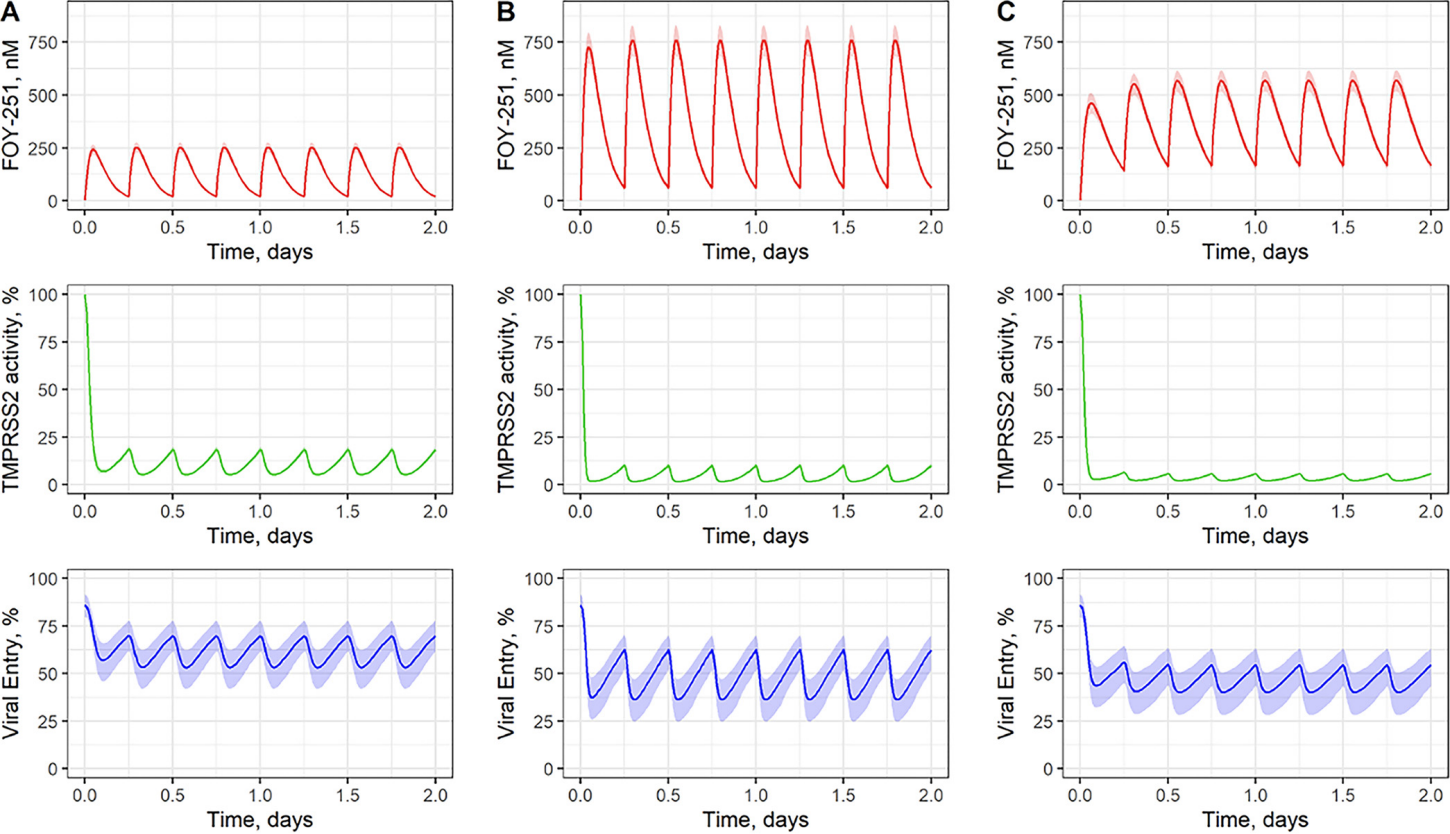
Predicted pharmacokinetic models, TMPRSS2 activity, and viral entry rate at different camostat doses. The PK model predictions (FOY-251 in plasma) are shown in the top panel by red solid lines with 90% CIs shown by filled bars. The predictions for TMPRSS2 activity (middle panel, green solid lines) and viral entry rate (bottom panel, blue solid lines) are shown with 90% CIs shown by filled bars. (A) camostat 200 mg q6h; (B) camostat 600 mg q6h; (C) camostat 600 mg q6h with two times slower.

The PK/PD model simulations were done for varying camostat oral doses (Fig. 3). The time averaged values of the model outcomes are listed in Table 3.

**TABLE 3:** Model predictions for different camostat mesylate doses^*a*^

Camostat dosing regimen	FOY-251 Cc_avg, nM	Avg TMPRSS2 activity, % (100% = enzyme activity at baseline; 0% = full enzyme inhibition)	Avg viral entry rate, % (100% = viral entry rate at baseline; 0% = complete viral entry rate inhibition)
Camostat 200 mg q8h (recommended dose)	96.6 (86.5, 105)	14.9 (13.6, 16.4)	64.2 (55.3, 71.9)
Camostat 200 mg q6h	129 (115, 139)	10.3 (9.15, 11.4)	59.9 (50.5, 68.3)
Camostat 400 mg q6h	258 (231, 279)	6.06 (5.34, 6.84)	51.5 (41.0, 60.9)
Camostat 600 mg q6h	386 (346, 418)	4.38 (3.84, 4.98)	46.4 (35.3, 56.0)
Camostat 600 mg q6h with a two times slower absorption rate	387 (346, 419)	3.44 (2.98, 3.93)	44.6 (33.0, 54.4)

a CIs 90% are given in parentheses.

The effect of the TMPRSS2 half-life on PK/PD model outcomes was explored. A notable increase of viral entry rate inhibition is predicted with enzyme half-life is increased from 4 h to 12 h. Similar viral entry rate inhibitions were calculated for enzyme half-life values of 12 h and 24 h (Fig. S3).

For the case where camostat is dosed 200 mg four times daily (QID), the model predicts 90% inhibition of TMPRSS2. Importantly, however, this translated to only about 40% inhibition of the viral entry rate were predicted. In the case where a camostat dose was increased 3-fold (600 mg QID), only a moderate improvement of inhibition of viral entry rate to 54% is predicted.

The effect of a drug slower absorption rate due to a hypothetical new formulation was also tested (Fig. 3). This scenario leads to smoother viral entry rate inhibition profile. Notably, a slower absorption rate of the drug also leads to smaller Cmax, that which might be important for safety considerations in the event such formulations are investigated.

To explore the drug exposure in ELF in more detail the PK/PD model was updated with epithelial lining fluid compartment (Fig. S2B). To describe FOY-251 PK in ELF airways, two unknown parameters were added—the drug transition rate between plasma and ELF compartments and the drug degradation rate in ELF compartment, which is hypothetically smaller than the drug elimination rate in central compartment. The PK/PD model simulations were done for four sets of parameter values describing the PK in ELF (rapid FOY-252 entry into the ELF then with either rapid or slow removal from the ELF and slow FOY-251 entry into the ELF with either rapid or slow removal from the ELF) (Fig. S4). A faster transition rate between compartments leads to larger exposure of the drug in ELF. The impact of the drug degradation rate in ELF might be also meaningful. The structure-based prediction supports a FOY-251 fast transition rate. The PK/PD simulations with fast transition rate of the drug (k_tr, elf_ =1.0 1/h) are shown in Fig. S4A and B, and demonstrate similar TMPRSS2 and viral entry rate inhibition in both central and ELF compartments.

## DISCUSSION

We present results from modeling the pharmacokinetics/pharmacodynamics of the serine protease inhibitor, camostat, interacting with its target, transembrane protease serine 2 (TMPRSS2), into which we incorporated the known and potential pharmacological mechanisms of the drug action. Based on the results of preclinical studies were reviewed in Breining et al. (28), a number of phase II clinical trials have been testing the repurposing of camostat for the treatment of COVID-19, both in inpatients with at least moderately severe disease and in outpatients with early infection/disease.

Human pharmacokinetic data has been published Midgley et al. (23) and *in vitro* data about anti-SARS-CoV-2 effect of camostat has been published separately (18). Here an integrated approach has been carried out using published data. The approach we present here may well be applied to other antiviral agents used in the COVID-19 disease state.

Camostat and its active metabolite FOY-251 are not only competitive inhibitors of TMPRSS2 and analogous serine proteases. Their enzyme inhibition occurs via covalent binding of serine residues in the TMPRSS2 active site (25, 26). The PK model here accounts for the prolonged on-rate and slow off-rate of FOY-251 covalently bound to TMPRSS2. The modeling predicts greater than 90% TMPRSS2 inhibition by conventional camostat dosing, but only partially inhibits viral entry rate. These modeling results are directly relevant to the future development of camostat in terms of delivery systems and molecular modification.

What are the strengths and weaknesses of this camostat pharmacodynamic modeling approach? Our PK model of camostat/FOY-251 in man allowed an excellent ability to simulate different camostat dosing regimens and predicted FOY-251 concentrations in plasma. Additionally, using the modeling concept originally applied to antibiotics (24), we were able to model FOY-251 exchange between plasma and ELF, an anatomical site in the lung that the SARS-CoV-2 certainly populates and drives the pulmonary pathology. Our simulations suggest only minimal to modest differences in the drug concentration profiles between plasma and ELF compartment. A limitation of our ELF modeling approach is that there are no known measurements of FOY-251 in pulmonary bronco-alveolar fluid samples which would approximate the ELF.

There are several implications of the camostat PK/PD modeling with relation to the treatment of COVID-19. On the other hand, there remain important, open questions. Will camostat sufficiently reduce the rate of viral cell entry sufficiently to improve clinical outcomes? How does camostat/FOY-251 antiviral efficacy depend on viral load? On which stage of COVID-19 would camostat treatment might be most effective? Are there host-associated targets of camostat that might improve clinical outcome independent of antiviral effects? To answer these questions the results of camostat clinical trials need to be conducted in which the SARS-CoV-2 viral load dynamics are measured simultaneously with detailed symptom scores and *ex vivo* biomarkers of protease-inhibitory effects. Virological endpoints by themselves may be insufficient, however, and must be combined with patient-oriented outcomes, including highly detailed quantification of the effect of camostat on the natural history of signs and symptoms of COVID-19. Key questions are timing of the camostat intervention, i.e., we need to understand at what time point in SARS-CoV-2 infection that inhibition of TMPRSS2 might affect the direction and path of infection. In addition to surrogate *in vitro* measures, biomarkers of improvement after early drug treatment of COVID-19 need to be established.

These concepts are directly relevant to the design of clinical studies for the repurposing of other drugs such as hydroxychloroquine and ivermectin, or for the design of new drugs *de novo* for the treatment and prevention of SARS-CoV-2/COVID-19. The additional mechanisms of SARS-CoV-2 S protein activation which are not linked with TMPRSS2 activity and camostat might be important in the antiviral drug effect. Prudence and a knowledge of history would suggest that a single antiviral agent approach to the treatment of COVID-19 may not be effective.

The present modeling study provides important insights into camostat therapeutics in man and supports further clinical development for COVID-19. Because respiratory tract viral load may be associated with negative outcome, even modestly reducing viral entry and respiratory tract viral load may reduce disease progression.

## MATERIALS AND METHODS

Experimental data from publicly available sources were digitized using Plot Digitizer version 2.5.0 software. Parameter and relative standard error estimation were based on the stochastic approximation expectation maximization (SAEM) algorithm and performed using Monolix software (Lixoft, Antony, France). Visualization of model diagnostics and simulations were performed in R software version 3.5.1, using the mlxR and ggplot2 library packages.

### Camostat/FOY-251 pharmacokinetic model.

A one-compartment PK model with linear elimination describes FOY-251 (the active metabolite of camostat) concentration profiles in human blood plasma. The model includes two routes of administration of camostat: intravenous and first order absorption from the gut with bioavailability coefficient F. Due to rapid transformation of camostat into FOY-251 *in vivo*, in the model camostat dose is considered as equimolar amount of FOY-251, i.e., camostat to FOY-251 transition was considered implicitly.

The ordinary differential equations (ODEs) for FOY-251 amount (in nmol) in gut (Ad) and central compartment (Ac): (Fig. 1B)

(1) $$\mathrm{dAd}/\mathrm{dt}=-k_{a}{}^{*}\mathrm{Ad}\;\;\;\;\;\;\;\;\;\;\;\;=k_{a}{}^{*}\mathrm{Ad}{}^{*}F-k_{\mathrm{el}}{}^{*}\mathrm{Ac}$$

Concentration of FOY-251 in blood plasma: Cc (nM) = Ac/V_d_.

The definition of the PK model parameters is given in Table 1.

### The approach to estimate FOY-251 exposure in ELF in airways.

One approach to estimate a drug exposure in ELF relative to exposure in plasma is to use drug molecular weight and octanol/water partition coefficient as key metrics (24). We used an empirical formula from (24) for this purpose:

(2) $$K=0.96+0.091\cdot \log\left(\mathrm{PC}\cdot {\mathrm{MW}}^{-1/2}\right)$$

where K is a ratio of drug concentration AUC in ELF to its AUC in plasma. PC is drug octanol-water partition coefficient and MW is molecular weight of the drug. FOY-251 MW = 313 g/mol and log(PC) theoretical estimates 0.6 and 1.63 were found (https://go.drugbank.com/metabolites/DBMET03117).

Using formula (2) and these two estimates of log(PC) for FOY-251 the values of K = 0.75 and 0.85 were obtained, respectively.

### TMPRSS2 specific activity inhibition by FOY-251 and link with SARS-CoV-2 entry efficiency inhibition.

*In vitro* experimental data were digitized from Hoffmann et al.’s Figure 4 (18) to link FOY-251 concentrations with recombinant TMPRSS2 activity inhibition.

The following ODEs were used to describe *in vitro* data of recombinant TMPRSS2 (SP) activity inhibition as function on time of incubation with FOY-251 concentration C (nM):

SP(t=0)=1(units)

SPi(t=0)=0

(3) $$\mathrm{dSP}/\mathrm{dt}=-k_{\mathrm{cat}}{}^{*}\mathrm{SP}{}^{*}C/\left(C+\mathrm{Ki}\right)+k_{\mathrm{dis}}{}^{*}\mathrm{SPi}$$

$$\mathrm{dSPi}/\mathrm{dt}=k_{\mathrm{cat}}{}^{*}\mathrm{SP}{}^{*}C/\left(C+\mathrm{Ki}\right)-k_{\mathrm{dis}}{}^{*}\mathrm{SPi}$$

where SP and SPi are amounts of TMPRSS2 molecules in active and inactive states, respectively.

An empirical Hill-Langmuir equation was used to link TMPRSS2 activity (SP) with SARS-2-S bearing pseudo-type viral particles entry rate, as measured *in vitro* in Hoffmann et al.’s Figure 7 (18).

(4) $$\mathrm{Viral Entry}=100\%\cdot {\mathrm{SP}}^{h}/\left({\mathrm{SP}}^{h}+K_{\mathrm{sp}}{}^{h}\right)$$

To make *in vivo* predictions of the drug effect the model was updated to include TMPRSS2 turnover.

Without drug TMPRSS2 turnover equation is:

$$\mathrm{dSP}/\mathrm{dt}=k_{\mathrm{synt}}-k_{\deg}\cdot \mathrm{SP}$$

using the assumption that at steady-state SP = 1 (unit) we obtain k_synt_ = k_deg_ · 1 (units/h).

The ODEs for TMPRSS2 inhibition by FOY-251 *in vivo*:

(5) $$\mathrm{dSP}/\mathrm{dt}=k_{\deg}\cdot \left(1-\mathrm{SP}\right)-k_{\mathrm{cat}}\cdot \mathrm{SP}\cdot \mathrm{Cc}/\left(\mathrm{Cc}+K_{i}\right)+k_{\mathrm{dis}}\cdot \mathrm{SPi}$$

$$\mathrm{dSPi}/\mathrm{dt}=-k_{\deg}\cdot \mathrm{SPi}+k_{\mathrm{cat}}\cdot \mathrm{SP}\cdot \mathrm{Cc}/\left(\mathrm{Cc}+K_{i}\right)-k_{\mathrm{dis}}\cdot \mathrm{SPi}$$

The TMPRSS2 catalytic domain is on the target cell surface exposed to extracellular fluid. Thus, considering fast exchange between plasma and extracellular fluid, the drug concentration in plasma (Cc) was used in Equation 5.

The definition of the PD model parameters is given in Table 2.

## Supplementary Material

Reviewer comments
