# Supplementary material for: Semi-Mechanistic Pharmacokinetic-Pharmacodynamic Model of Camostat Mesylate-Predicted Efficacy against SARS-CoV-2 in COVID-19
Source: Microbiol Spectr. 2022 Apr 12;10(2):e02167-21. doi: 10.1128/spectrum.02167-21 (PMC9047529; doi:10.1128/spectrum.02167-21)
Supplement: SUPPLEMENTAL FILE 1 — Supplemental material. Download spectrum.02167-21-s001.pdf, PDF file, 0.7 MB [file spectrum.02167-21-s001.pdf]

SUPPLEMENTARY MATERIALS

Figure 1S. Camostat/FOY-251 PK model (solid lines) fits FOY-251 concentration measurements (dots) in plasma for A: Camostat 40 mg i.v. infusion over 12 h (data from Midgley et al. 1994) and B: Camostat 200 mg orally single dose (data from FOIPAN© package insert).

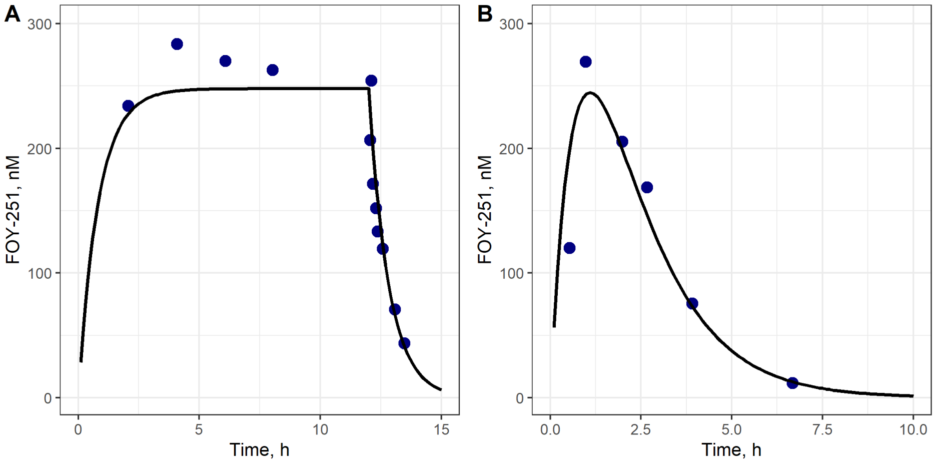

Figure 2S. FOY-251 PK model extension with Epithelial Lining Fluid (ELF) compartment. (A) Schematic diagram of the blood-alveolar drug barrier (adopted from Kiem & Schentag, 2008) (B) The PK model scheme.

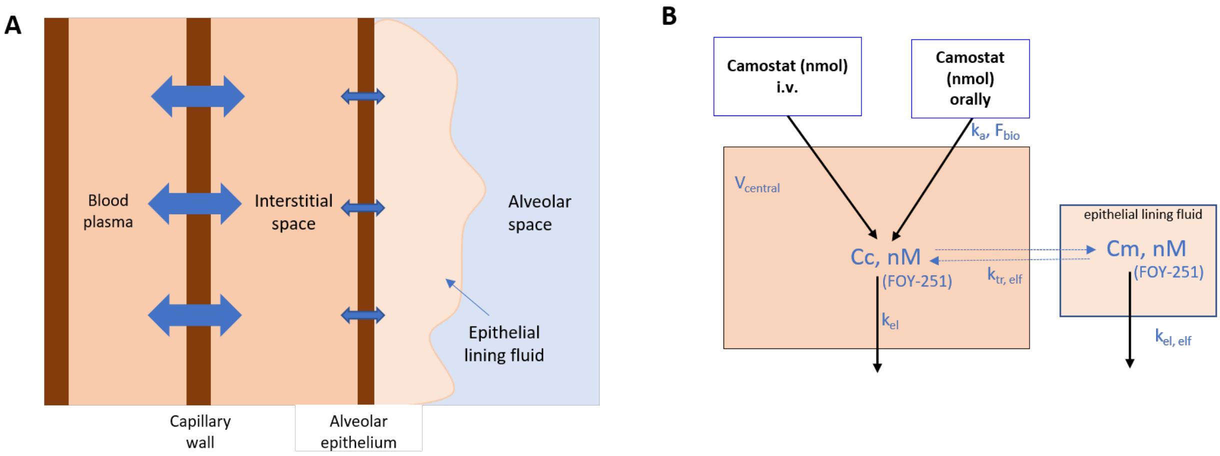

The PK in central compartment is assumed to be independent on ELF compartment.

To describe the drug concentration profile in ELF (or mucosa) compartment we use the equation:

$$dCm/dt = k_{tr\_elf} \cdot (Cc - Cm) - k_{el\_elf} \cdot Cm$$

There are two unknown parameters in the equation:

k_{tr_elf} (1/h) – FOY-251 transition rate through the bronchial/alveolar epithelium. FOY-251 is small water-soluble molecule with minor binding to plasma proteins, and predicted transition rate should be relatively fast (S.Kiem and J.J.Schentag 2008 Antimicrobial Agents and Chemotherapy).

k_{el_elf} (1/h) - FOY-251 degradation rate in ELF compartment, hypothetically, smaller than k_{el} in central compartment.

Figure 3S. The impact of TMPRSS2 (active and inhibited by FOY-251) *in vivo* half-life. Camostat 200 mg q6h PKPD were simulated under assumption about TMPRSS2 half-life in vivo: A: 4 h; B: 12 h; C: 24 h. Top panel: The PK model predictions (FOY-251 in plasma) are shown by red solid lines with 90% CIs shown by filled bars. Middle panel: predictions for TMPRSS2 activity are shown by green solid lines with 90% CIs shown by filled bars. Bottom panel: Viral entry rate predictions are shown by blue solid lines with 90% CIs shown by filled bars.

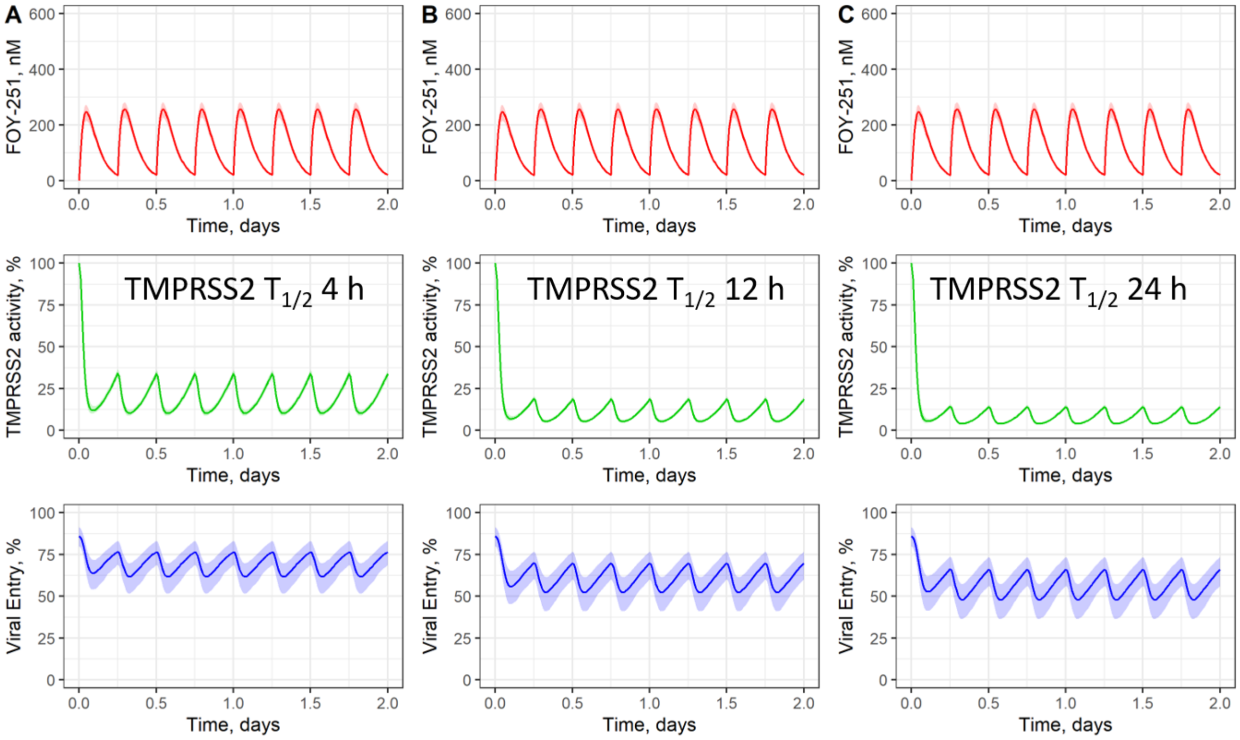

Figure 4S FOY-251 PKPD model extension with Epithelial Lining Fluid (ELF) compartment. The PKPD model simulations. Camostat 200 mg q6h PKPD were simulated under different assumptions about FOY-251 plasma-ELF transition rate and FOY-251 degradation rate in ELF compartment. A: k_{tr_elf} = 1.0 1/h and k_{tr_elf} = 0.2 · k_{el_plasma} (rapid ELF entry, slow removal); B: k_{tr_elf} = 1.0 1/h and k_{tr_elf} = 0.5*kel_plasma (rapid ELF entry, rapid removal); C: k_{tr_elf} = 0.2 1/h and k_{tr_elf} = 0.2 · k_{el_plasma} (slow ELF entry, slow removal); D: k_{tr_elf} = 0.2 1/h and k_{tr_elf} = 0.5 · k_{el_plasma} (slow ELF entry, rapid removal). The PKPD model predictions in plasma compartment are shown by solid lines with 90% CIs shown by filled bars. The predictions in ELF compartment are shown by dashed lines.

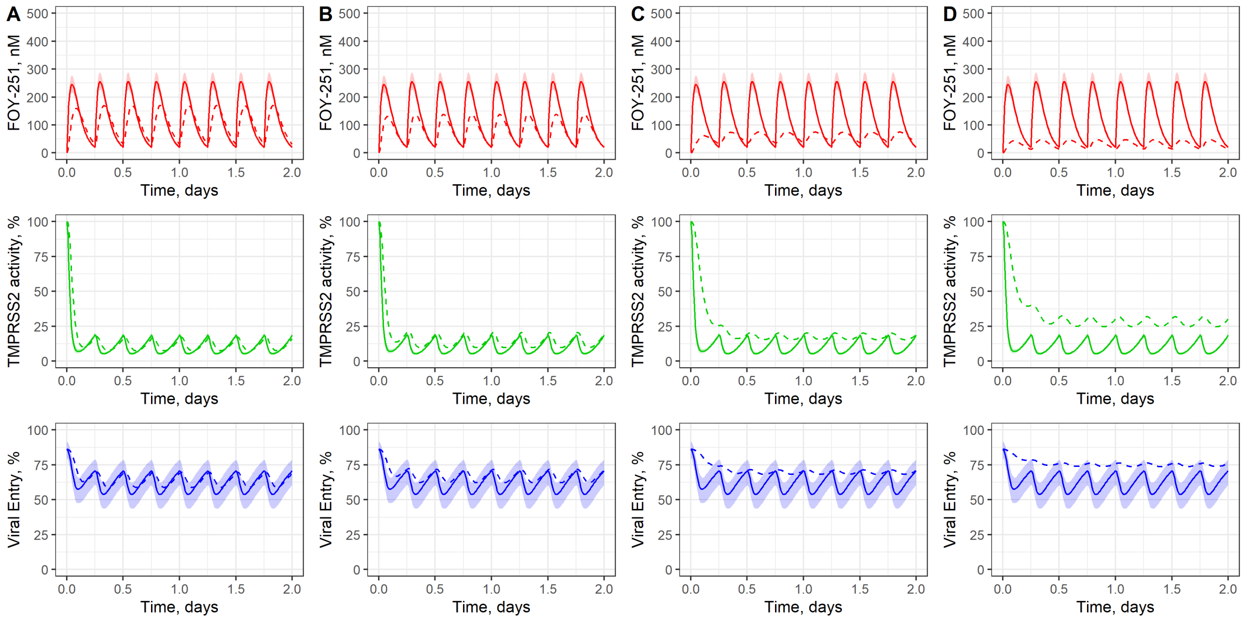

References

Midgley I, Hood AJ, Proctor P, et al. Metabolic fate of ^{14}C-camostat mesylate in man, rat and dog after intravenous administration. **1994**; Xenobiotica 24: 79-92.

Kiem S, Schentag JJ. Interpretation of antibiotic concentration ratios measured in epithelial lining fluid. **2008**; Antimicrob Agents Chemother 52: 24-36.
